# *Paracoccidioides brasiliensis* downmodulates α3 integrin levels in human lung epithelial cells in a TLR2-dependent manner

**DOI:** 10.1038/s41598-020-76557-6

**Published:** 2020-11-10

**Authors:** Bianca Carla Silva Campitelli de Barros, Bruna Rocha Almeida, Erika Suzuki

**Affiliations:** Department of Microbiology, Immunology, and Parasitology, Escola Paulista de Medicina, Universidade Federal de São Paulo, São Paulo, 04023-062 Brazil

**Keywords:** Fungi, Pathogens, Cell biology, Cell signalling

## Abstract

Paracoccidioidomycosis (PCM) is the most prevalent systemic mycosis in Latin America and may be caused by the species *Paracoccidioides brasiliensis*. In the lungs, this fungus interacts with epithelial cells, activating host cell signalling pathways, resulting in the production of inflammatory mediators. This event may be initiated through the activation of Pattern-Recognition Receptors such as Toll-like Receptors (TLRs). By interacting with cell wall components, TLR2 is frequently related to fungal infections. In this work, we show that, after 24 h post-infection with *P. brasiliensis,* A549 lung epithelial cells presented higher TLR2 levels, which is important for IL-8 secretion. Besides, integrins may also participate in pathogen recognition by host cells. We verified that *P. brasiliensis* increased α3 integrin levels in A549 cells after 5 h of infection and promoted interaction between this receptor and TLR2. However, after 24 h, surprisingly, we verified a decrease of α3 integrin levels, which was dependent on direct contact between fungi and epithelial cells. Likewise, we observed that TLR2 is important to downmodulate α3 integrin levels after 24 h of infection. Thus, *P. brasiliensis* can modulate the host inflammatory response by exploiting host cell receptors and cell signalling pathways.

## Introduction

Paracoccidioidomycosis (PCM) is a deep human mycosis that is restricted to Latin America^1^ and affects commonly male rural workers. Although this disease has been frequently related to poverty, unfortunately, it is still not recognised as a neglected tropical disease by the World Health Organisation (WHO)^2^. Nowadays, environmental factors are changing PCM epidemiology, which is reflected in the increasing number of patients in some regions^3–5^. One example was recently reported by Do Valle and co-workers (2017), who described the increase of PCM cases by sixfold after a highway construction in Rio de Janeiro (Brazil), possibly caused by deforestation and the removal of vast amounts of soil^5^.

PCM is caused by dimorphic fungi from the genus *Paracoccidioides* that present mycelial and yeast forms. Host infection may occur by inhalation of mycelial propagules, probably contained in contaminated soil, and once in the lungs, these fungi convert into yeasts^1^. Aiming to survive, pathogens adopt some mechanisms to exploit host cells, leading to the establishment, maintenance and dissemination of the infection.

Epithelial cell is an important cell type of host lungs that interacts with inhaled pathogens, not only primarily forming a physical barrier against microorganisms, but also acts in the innate immune response by secreting inflammatory mediators such as cytokines and chemokines, which results in the recruitment of immune cells to the site of infection^6^.

Over the past years, our research group has focused on studying the lung epithelial cell responses to fungal infections, especially regarding the mechanisms involved in the release of inflammatory cytokines induced by yeasts of *Paracoccidioides* or *Histoplasma capsulatum*^7–13^. Our data have demonstrated that different fungal species promote cytokine secretion in lung epithelial cell in distinct manners. In fact, we verified that the stimulation mechanism of cytokine release differs even among isolates of the same species^12,13^.

It was also observed that *P. brasiliensis* yeasts interact with α3 and α5 integrins in A549 epithelial cells and promote the recruitment of these receptors to host cell structures known as lipid rafts. In addition, we verified that both integrins and these lipid platforms are involved in IL-8 secretion by *P. brasiliensis* infected-epithelial cells^9^.

Besides integrins, other receptors such as Toll-like Receptors (TLRs) have been extensively studied in fungal infections^14,15^. Under particular stimuli, TLRs may also collaborate with integrins to activate signalling pathways that culminate in cytokine secretion^16,17^. Marre et al., for example, verified that the TLR2 synthetic peptide ligand PAM_3_CSK_4_ induces cooperation between the heterodimers TLR2/1 and α3β1 integrin in macrophages, which results in IL-6 secretion by these cells^16^.

Evidence that lung epithelial cells influence host immune responses against infections is increasing over the past decade^6^. In the present work, we investigated the involvement of TLR2 in IL-8 secretion that was induced by *P. brasiliensis* yeasts in lung epithelial cells. Moreover, we analysed whether TLR2 interacts with α3 integrin and modulates its expression in A549 cells during this fungal infection.

## Results

### TLR2 expression levels in lung epithelial cells infected with *P. brasiliensis* yeasts, and involvement of this receptor in IL-8 secretion

**Figure 1:**
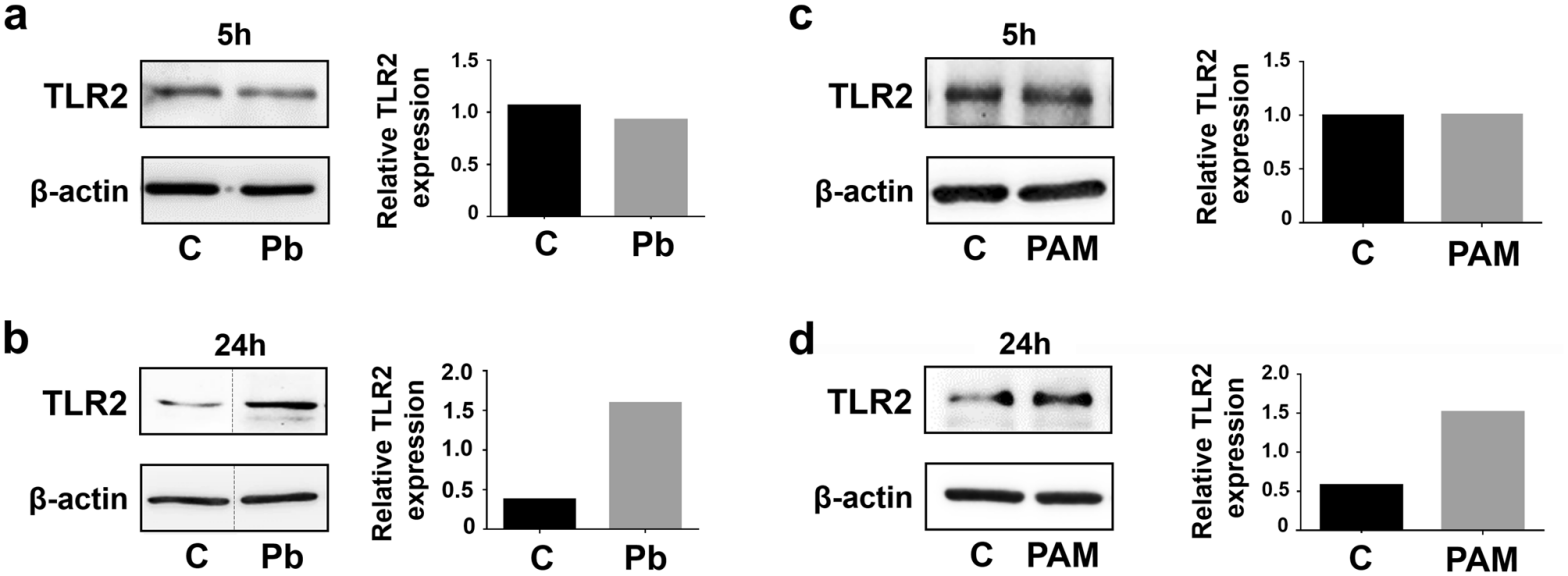
TLR2 levels in A549 cells incubated with *P. brasiliensis* yeasts or PAM_3_CSK_4_. (**a**,**b**) A549 cells were incubated or not (C) with *P. brasiliensis* yeasts (MOI = 2.5) (Pb). (**c**,**d**) A549 cells were incubated or not (C) with 5 μg/mL of the TLR2-specific ligand PAM_3_CSK_4_ (PAM). After 5 h or 24 h, epithelial cells were lysed and aliquots were submitted to SDS–PAGE. TLR2 expression was analysed by Western blot. β-actin was used as loading protein control. Relative TLR2 levels were determined by densitometric analysis of the bands. Values represent the ratio of the intensity of TLR2 band divided by the corresponding intensity of β-actin band. Similar results were obtained from three independent experiments. Dashed lines in (**a**,**b**) represent cropped images. Uncropped images are presented in Supplementary Fig. S1.

After incubation of A549 epithelial cells with *P. brasiliensis* yeasts (isolate Pb 18), we evaluated by Western blot whether this fungus modulates the expression of TLR2. Figure 1a shows that, after 5 h of fungal infection, TLR2 levels of epithelial cells were very similar to uninfected cells. However, after 24 h of A549-*P. brasiliensis* interaction, we verified higher TLR2 levels (3.55-fold) than A549 cell basal levels (Fig. 1b).

**Figure 2:**
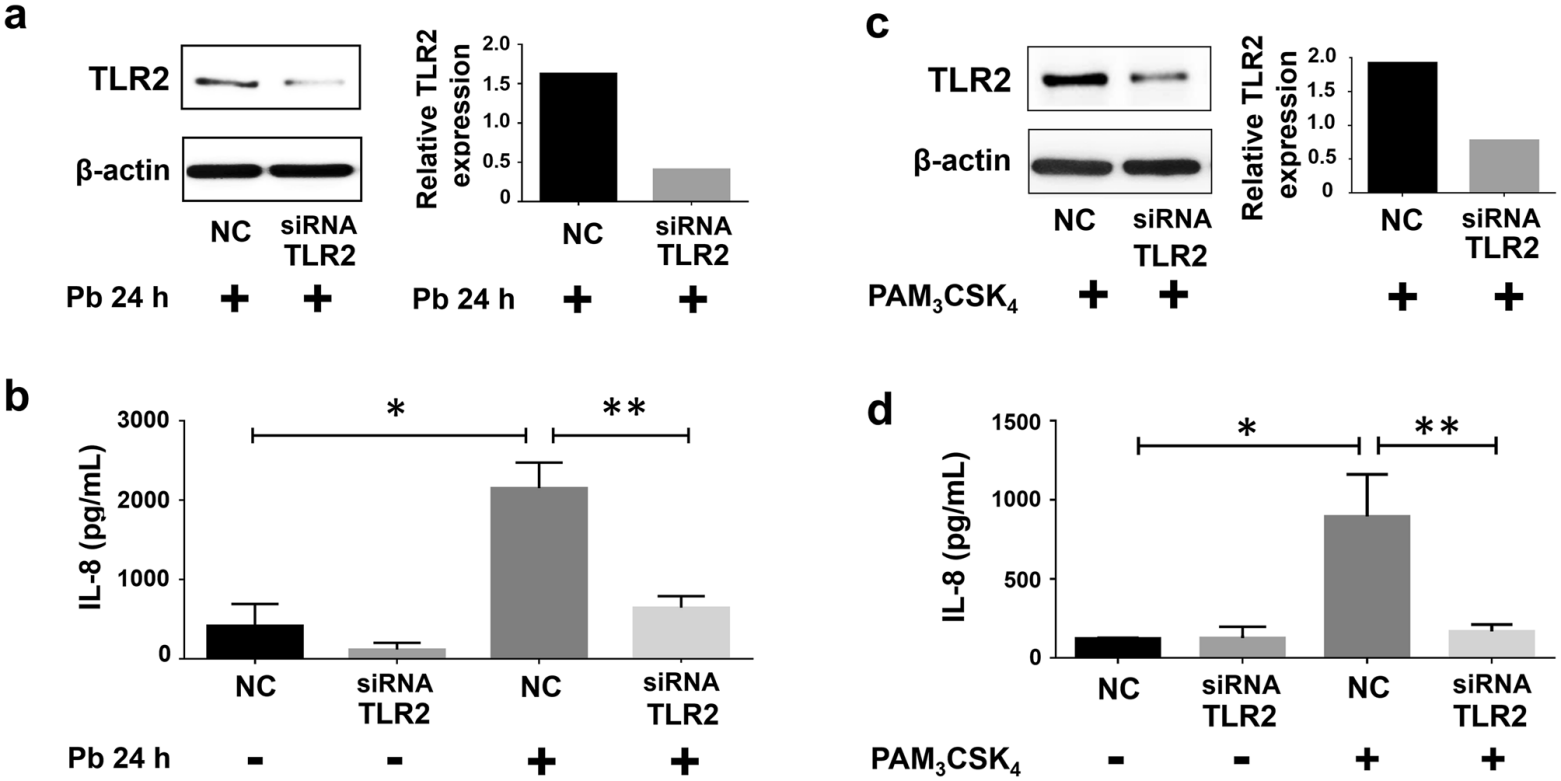
Effect of TLR2 silencing on IL-8 secretion by A549 cells incubated with *P. brasiliensis* yeasts or PAM_3_CSK_4_. A549 cells were transfected with siRNA directed to TLR2 (siRNA TLR2) or negative control siRNA (NC), and then incubated with *P. brasiliensis* yeasts (MOI = 2.5) (Pb 24 h +) or with 5 μg/mL of the TLR2-specific ligand PAM_3_CSK_4_. After 24 h, epithelial cells were lysed and aliquots were submitted to SDS–PAGE and Western blot, using anti-TLR2 antibodies (**a**,**c**). β-actin was used as loading protein control. Relative TLR2 levels were determined by densitometric analysis of the bands (**a**,**c**). (**b**,**d**) Cell supernatants were collected, and IL-8 levels of siRNA-transfected A549 cells, during incubation with *P. brasiliensis* yeasts or with PAM_3_CSK_4_, were analysed by ELISA. Values represent the mean of triplicate experiments ± standard deviation. *, *p* < 0.01 when compared to A549 cells transfected with NC in the absence of *P. brasiliensis* or PAM_3_CSK_4_. **, *p* < 0.01 when compared to A549 cells transfected with NC and incubated with *P. brasiliensis* yeasts or PAM_3_CSK_4_. Uncropped images are presented in Supplementary Fig. S2.

Next, we analysed whether secretion of IL-8 by A549 cells, promoted by *P. brasiliensis*, is TLR2-dependent. For this, epithelial cells were transfected with negative control (NC)- or with TLR2-directed small interfering RNA (siRNA), and then, infected with *P. brasiliensis* yeasts. After 24 h, IL-8 concentrations were measured by ELISA. As expected, this fungus induced an increase of this chemokine levels by 5.2-fold when compared to uninfected cells (Fig. 2b, samples NC). When TLR2 was silenced, we verified a reduction of 70% of IL-8 secretion when compared to *P. brasiliensis*-infected NC cells (Fig. 2b), indicating that this fungus promotes release of this cytokine in a TLR2-dependent manner.

To confirm that A549 cells are able to secrete IL-8 through TLR2 cell signalling pathway, we incubated A549 cells with the ligand PAM_3_CSK_4_, which specifically interacts with TLR2/1 heterodimer^18,19^. First, we observed higher TLR2 levels than control cells only after 24 h incubation of epithelial cells with PAM_3_CSK_4_ (Fig. 1c,d). Then, we analysed IL-8 secretion by A549 cells after incubation with this peptide. As expected, PAM_3_CSK_4_ promoted an increase of IL-8 levels by 7.3-fold when compared to levels of NC cells without this ligand. Moreover, in PAM_3_CSK_4_-stimulated A549 cells, TLR2-directed siRNA reduced IL-8 secretion by 81.3% when compared to PAM_3_CSK_4_-NC cell cultures (Fig. 2d), corroborating that A549 epithelial cells may secrete chemokine IL-8 in a TLR2 dependent manner.

By Western blot, efficiency of TLR2 silencing was analysed, and we verified that, when compared to transfected NC cells, the levels of this receptor were decreased by 75% or 59.1% in TLR2-siRNA transfected A549 cells incubated with *P. brasiliensis* yeasts or PAM_3_CSK_4_**,** respectively (Fig. 2a, c).

### *Paracoccidioides brasiliensis* increases interaction between α3 integrin and TLR2 in A549 cells

**Figure 3:**
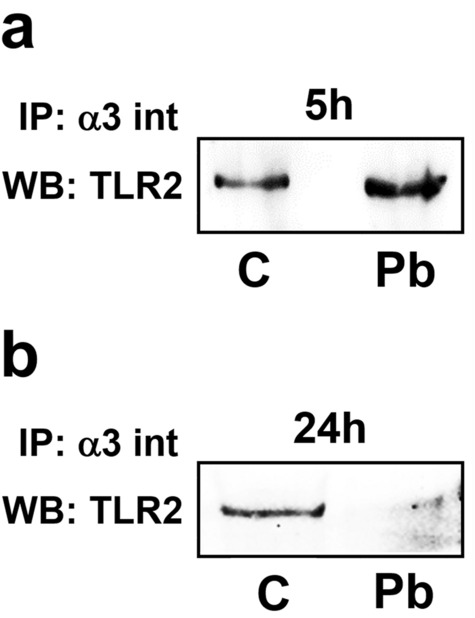
Interaction of α3 integrin with TLR2 in A549 cells infected with *P. brasiliensis* yeasts. A549 cells were incubated (Pb) or not (C) with *P. brasiliensis* yeasts (MOI = 2.5). After 5 h (**a**) or 24 h (**b**), cells were lysed and samples were incubated with anti-α3 integrin (α3 int) antibodies (IP). After 16 h, agarose beads conjugated with A/G protein were added. Then, sample buffer was added, and the resultant supernatant was submitted to SDS–PAGE. TLR2 was analysed by Western blot (WB). Similar results were obtained from three independent experiments. Uncropped images are presented in Supplementary Fig. S3.

Since *P. brasiliensis* yeasts induce IL-8 secretion in A549 cells in α3 integrin-^9^ and TLR2-dependent manners (Fig. 2b), we analysed whether these two receptors interact with each other during fungal infection. For this, after *P. brasiliensis*-A549 cell incubation, α3 integrin was immunoprecipitated and TLR2 was detected by Western blot. Figure 3a shows an increase of the co-immunoprecipitated α3 integrin-TLR2 complexes, obtained from A549 epithelial cells incubated for 5 h with *P. brasiliensis* yeasts, when compared to uninfected cells. On the other hand, after 24 h *P. brasiliensis*-A549 cell infection, we did not observe α3 integrin-TLR2 complexes (Fig. 3b). This last result was unexpected for us, since 24 h-infected A549 cells presented notable TLR2 protein levels (Fig. 1b).

### *Paracoccidioides brasiliensis* yeasts modulate α3 integrin levels in epithelial cells

**Figure 4:**
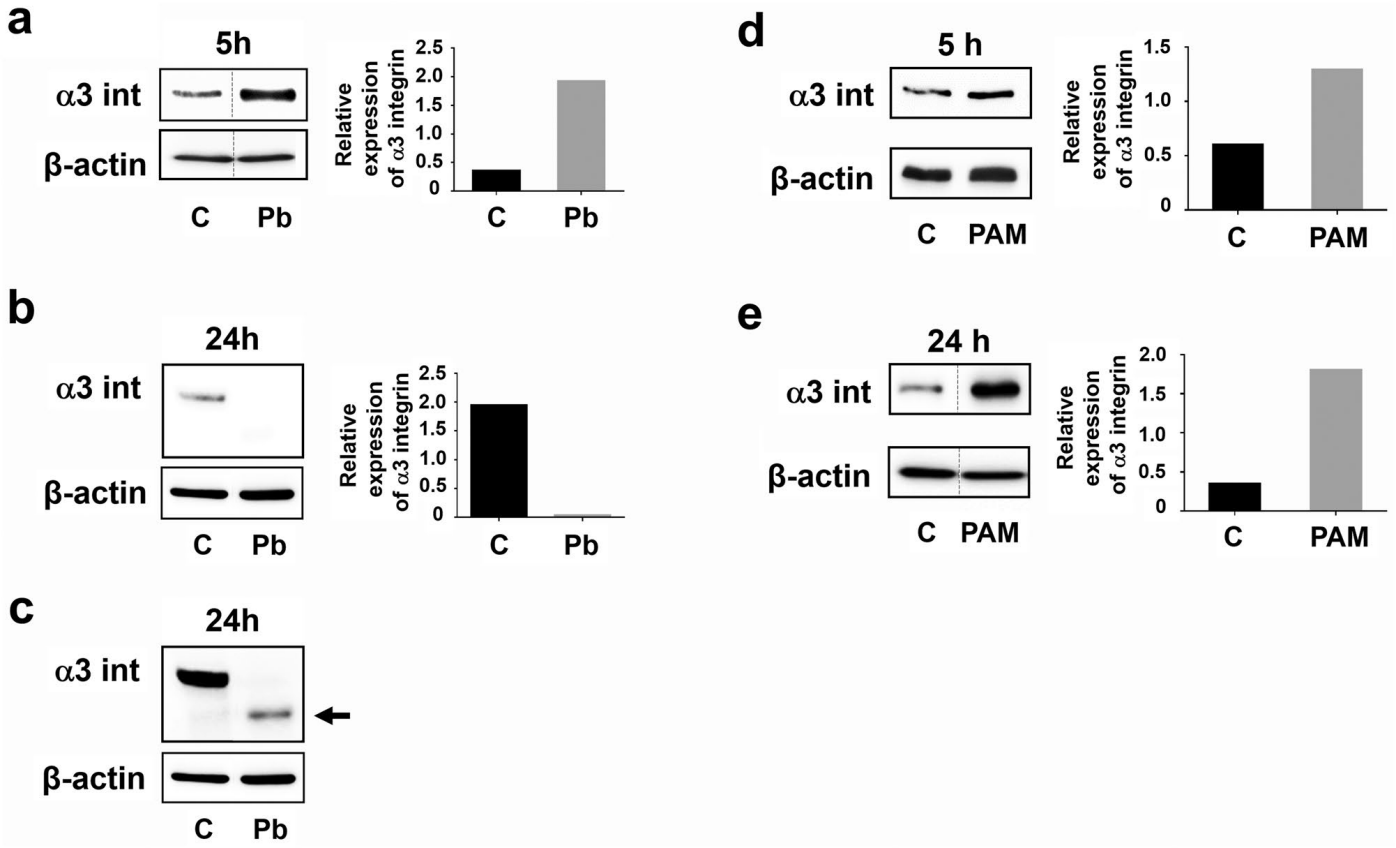
α3 integrin levels in A549 cells after incubation with *P. brasiliensis* yeasts or PAM_3_CSK_4_. A549 cells were incubated or not (C) with *P. brasiliensis* yeasts (Pb) (MOI = 2.5) (**a–c**) or with 5 μg/mL of the TLR2-specific ligand PAM_3_CSK_4_ (PAM) (**d**,**e**)_**.**_ After 5 h or 24 h, epithelial cells were lysed and aliquots were submitted to SDS–PAGE. α3 integrin levels (α3 int) were analysed by Western blot. (**c**) α3 integrin levels from (**b**) in a higher exposure time in the UVITEC imaging system. Arrow corresponds to a band with lower molecular weight than α3 integrin (150 kDa). β-actin was used as loading protein control. Relative α3 integrin levels were determined by densitometric analysis. Values represent the ratio of the intensity of α3 integrin band divided by the corresponding intensity of β-actin band. Similar results were obtained from three independent experiments. Dashed lines in **(a**,**e)** represent cropped images. Uncropped images are presented in Supplementary Fig. S4.

Next, we investigated α3 integrin protein levels in infected-A549 cells after 5 h and 24 h. Corroborating previous data^9^, *P. brasiliensis* yeasts induced an increase (5.4-fold) of α3 integrin levels in A549 cells after 5 h incubation (Fig. 4a). However, after 24 h, this integrin levels decreased by 98.3% in *P. brasiliensis*-infected A549 cells (Fig. 4b). Longer exposure time of this PVDF membrane enabled to detect a band, recognised by an anti-α3 integrin antibody. This band (Fig. 4c, arrow) presented a lower molecular weight than α3 integrin (150 kDa), suggesting a degradation of this receptor that was induced by *P. brasiliensis* yeasts in A549 epithelial cells.

In addition, we verified whether the TLR2-specific ligand PAM_3_CSK_4_ could modulate α3 integrin levels in A549 cells. As shown in Fig. 4d, after 5 h of incubation, PAM_3_CSK_4_ increased α3 integrin levels by two fold when compared to basal levels, and by 5.2-fold when the peptide was incubated for 24 h with epithelial cells (Fig. 4e), indicating that PAM_3_CSK_4_ can upmodulate α3 integrin expression.

**Figure 5:**
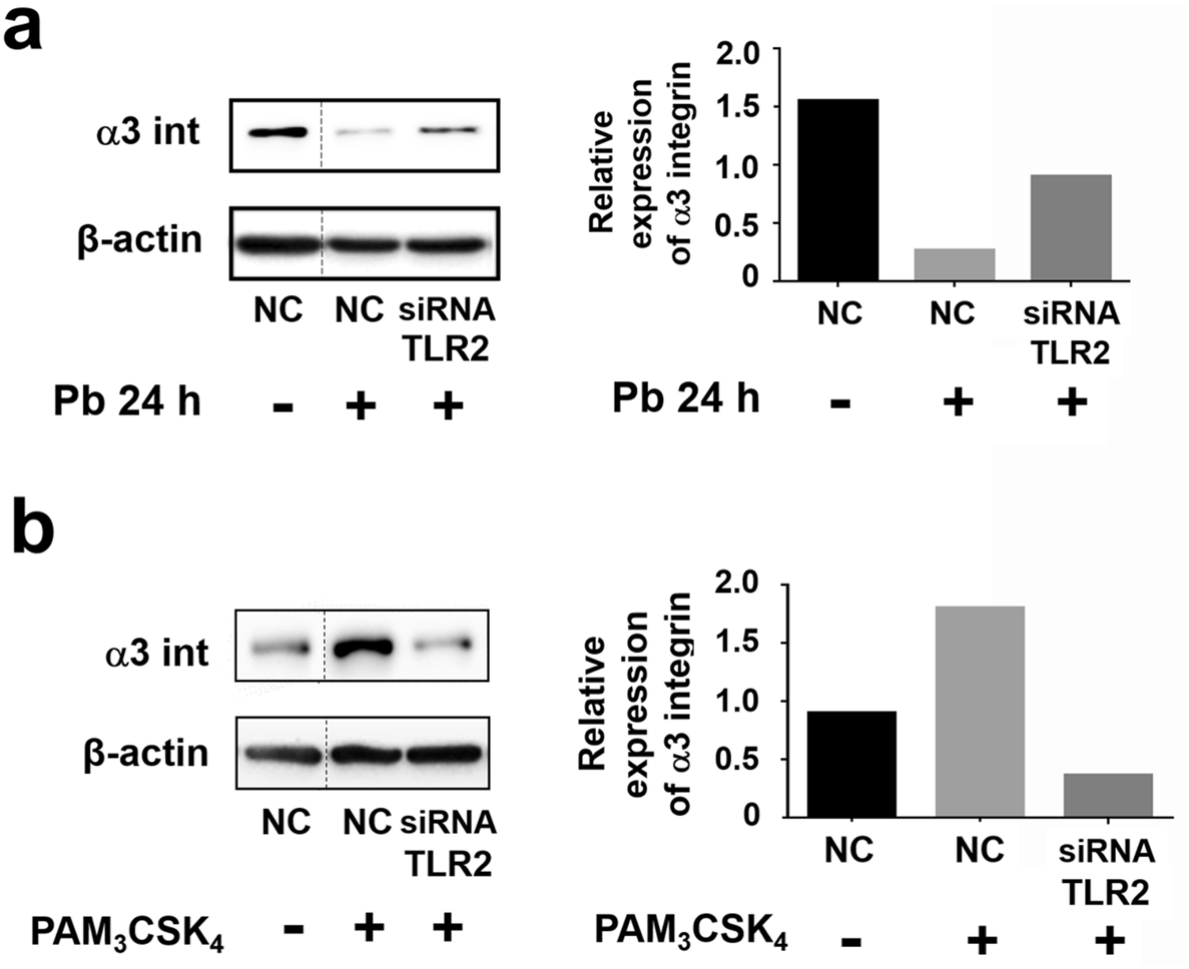
α3 integrin levels in TLR2-silenced A549 cells incubated with *P. brasiliensis* yeasts or PAM_3_CSK_4_. A549 cells were transfected with siRNA directed to TLR2 (siRNA TLR2) or negative control siRNA (NC). Next, cells were incubated with *P. brasiliensis* yeasts (MOI = 2.5) (Pb) (**a**) or with 5 μg/mL of the TLR2-specific ligand PAM_3_CSK_4_ (**b**). After 24 h, epithelial cells were lysed and aliquots were submitted to SDS–PAGE. α3 integrin levels (α3 int) were analysed by Western blot. β-actin was used as loading protein control. Relative α3 integrin levels were determined by densitometric analysis. Values represent the ratio of the intensity of integrin band divided by the corresponding intensity of β-actin band. Similar results were obtained from three independent experiments. Dashed lines in (**a,b**) represent cropped images. Uncropped images are presented in Supplementary Fig. S5.

As we found that *P. brasiliensis* yeasts downmodulate α3 integrin levels in A549 epithelial cells (Fig. 4b, 4c), we evaluated whether TLR2 may participate in this process. Therefore, after silencing this PRR by siRNA, A549 cells were incubated with *P. brasiliensis* for 24 h, and then, we analysed α3 integrin levels. Under these conditions, we observed that α3 integrin levels were 3.4-fold higher in TLR2-silenced cells than in *P. brasiliensis* infected-NC cells (Fig. 5a), indicating that downmodulation of this integrin occurs in a TLR2-dependent manner.

As shown in Fig. 5b, when compared to PAM_3_CSK_4_ incubated-NC cells, we observed that TLR2-siRNA promoted a reduction of α3 integrin levels up to 80%. In this manner, unlike *P. brasiliensis*, PAM_3_CSK_4_ upmodulates α3 integrin levels and TLR2 is also important for this process.

**Figure 6:**
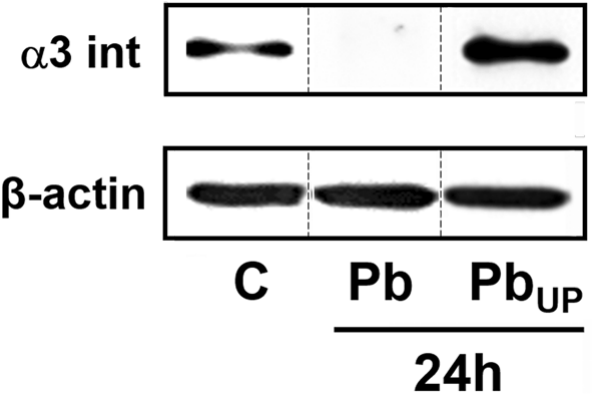
α3 integrin levels in A549 cells infected with *P. brasiliensis* yeasts using a Transwell system. A549 cells were incubated with *P. brasiliensis* yeasts (MOI = 2.5), which were in direct contact with epithelial cells (Pb) or disposed in the upper compartment of a Transwell system (Pb_UP_). Control (C) was performed in the absence of yeasts. After 24 h, epithelial cells were lysed and aliquots were submitted to SDS–PAGE. α3 integrin levels were analysed by Western blot. β-actin was used as loading protein control. Similar results were obtained from three independent experiments. Dashed lines represent cropped images. Uncropped images are presented in Supplementary Fig. S6.

As *P. brasiliensis* secretes compounds that may promote different effects in the host^11,12^, we verified if the decrease of α3 integrin levels in A549 cells after 24 h could be a result of this fungal secretion. For this, A549 cells were cultured in 6-well plates, and *P. brasiliensis* yeasts were placed in the upper compartment of Transwell platforms or in direct contact with the epithelial cells. As expected, *P. brasiliensis* incubated in direct contact with A549 cells promoted reduction of α3 integrin levels by 97.3% after 24 h (Fig. 6, Pb). However, when fungi were arranged in the Transwell upper compartment and, consequently, without contact with A549 cells, we observed a 2.8-fold increase in α3 integrin levels (Fig. 6, Pb_UP_). Thus, the direct contact between A549 cells and *P. brasiliensis* is mandatory for a yeast-induced reduction of α3 integrin levels in epithelial cells. Moreover, in a different manner, secreted factors by this fungus promoted an increase of α3 integrin levels in A549 cells.

## Discussion

In an infection process, inside the host, a pathogen may interact with a particular cell, engaging several receptors and highjacking host cell signalling pathways in order to survive. One family of receptors extensively studied in infection models is the Toll-Like Receptor (TLR) family. In fungal infections, by promoting the secretion of cytokines and chemokines, these receptors are important for the host immune response, recognising several ligands in these pathogens, such as O-linked mannans, phospholipomannans, and unmethylated DNA with CpG motif ^20,21^. In the present study, we demonstrated that *P. brasiliensis* modulates TLR2 expression in A549 epithelial cells and, by silencing this receptor, we also found that TLR2 participates in IL-8 secretion by these cells. In fact, some groups described the participation of TLR2 in the immune response against *P. brasiliensis*^22–24^. For example, the work developed by Balderramas and co-workers (2014) showed that *P. brasiliensis* binds to TLR2 to induce the production of IL-12 by neutrophils^23^. Another group, Loures and co-workers (2009), showed that TLR2 is an important receptor for the innate immune system in a *P. brasiliensis* infection, and also this receptor may have a protective effect in pulmonary fungal infections^24^.

In the literature, several groups have shown that TLRs and integrins pathways communicate with each other in different cell processes^16,17,20,25–27^. McGarry and co-workers (2015)^27^, for example, demonstrated that the TLR2 ligand PAM_3_CSK_4_ induces cell migration of the rheumatoid arthritis synovial fibroblast cells (RASFC), and also increases β1 integrin levels in these cells. Moreover, these authors observed that RASFC cell migration, induced by PAM_3_CSK_4_, was inhibited by β1 integrin neutralizing antibodies, demonstrating that activation of TLR2 induces RASFC migration that occurs in a β1 integrin dependent manner.

In the present work, we verified in A549 lung epithelial cells that the TLR2 ligand PAM_3_CSK_4_ increased α3 integrin levels, which was inhibited by silencing TLR2, both data indicate that the heterodimer TLR2/1 upmodulates this integrin levels in A549 epithelial cells. However, when these epithelial cells were infected with *P. brasiliensis* yeasts for 24 h, we observed higher levels of TLR2 and also that α3 integrin was almost undetectable by Western blot. The latter result was not expected since, in a previous study, we verified an augmentation of the expression of α3 and α5 integrins in A549 epithelial cells infected with this fungus for 5 h^9^. Regarding IL-8 secretion, although we observed the decrease of α3 integrin levels after 24 h of incubation with *P. brasiliensis* yeasts, the upmodulation of α3 integrin levels in the first 5 h of A549 cell infection seems to be important to promote this cytokine secretion, since after α3 integrin silencing, we observed a reduction of IL-8 levels^9^.

In summary, this work shows that during early infection (5 h), *P. brasiliensis* yeasts induce a raise in α3 integrin levels in A549 epithelial cells, while TLR2 levels are similar to those of uninfected cells. Moreover, interaction of α3 integrin with TLR2 occurs during this early period. However, in longer periods of infection (24 h), *P. brasiliensis* downmodulates α3 integrin levels in direct contact- and TLR2-dependent manners. Together, these results indicate that *P. brasiliensis* yeasts differently modulate the activity of host cell receptors and signalling pathways during the course of the infection. Interestingly, Kim and co-workers (2009)^28^ described that α3β1 integrin is involved in the formation of myofibroblasts and lung fibrosis. These authors showed that mice lacking α3 integrin-pulmonary epithelial cells, upon bleomycin stimulus, presented lower accumulation of myofibroblasts and did not progress to lung fibrosis. Moreover, lack of lung fibrosis is seen in acute cases of PCM^29^, a severe form of this mycosis. In this manner, the reduction of α3 integrin in lung epithelial cells promoted by *P. brasiliensis* might contribute to the pathogenesis of PCM acute form. Therefore, a better understanding of the cellular mechanisms that promote fungal infection will lead to the development of new therapies in a scenario where there are no vaccines against fungal infections, antifungal drugs are limited, and some fungi are already developing resistance to these drugs^20^.

## Methods

### Epithelial cell culture

A549 epithelial cell line (human lung adenocarcinoma) was cultured in Dulbecco’s Modified Eagle’s Medium—DMEM (Sigma-Aldrich/Merck, MO, USA) with 10% fetal bovine serum – FBS (Vitrocell, SP, Brazil), 10 mM HEPES, 100 U/mL penicillin, and 100 μg/mL streptomycin (Sigma-Aldrich/Merck, MO, USA) (complete DMEM) at 37 °C, in 5% CO_2_ atmosphere. For *P. brasiliensis*-A549 interaction assays, cells were grown in 100 mm^2^ dishes, 6-well or 24-well plates.

### *Paracoccidioides brasiliensis* yeast culture and preparation for interaction assays with epithelial cells

*P. brasiliensis* yeasts, Pb18 isolate, were kindly provided by Dr. Wagner Luiz Batista, from Universidade Federal de São Paulo (Diadema, SP, Brazil). Fungi were cultured in PGY medium (5 g/L neopeptone, 5 g/L yeast extract, 15 g/L glucose—Becton Dickinson, NJ, USA) containing 1.4 g/L asparagine and 0.1 g/L thiamine (Sigma-Aldrich/Merck, MO, USA) for 5–7 days in incubator shaker at 37 °C, 120 rpm, as previously described by Ywazaki and co-workers (2011) ^30^. Fungal virulence was maintained by reisolating *P. brasiliensis* from infected B10.A mice. For epithelial cell-fungus interaction assays, single fungal mother and daughter yeasts from *P. brasiliensis* were obtained as previously described by Barros and co-workers (2016) ^9^. The resultant supernatant contained only single fungal cells, which were washed three times with DMEM.

### Interaction between epithelial cells and *P. brasiliensis* yeasts

A549 epithelial cells were seeded in tissue culture plates. After 48 h, cells were washed three times with FBS-free DMEM, and incubated in this medium overnight, at 37 °C, 5% CO_2_ (starving). A549 cells were then incubated for 5 h or 24 h with: (1) 5 µg/mL of TLR2/1 ligand PAM_3_CSK_4_ (InvivoGen, CA, USA); or (2) *P. brasiliensis* yeasts with a multiplicity of infection (MOI) of 2.5 fungi for 1 epithelial cell. Controls without stimuli were also performed. Then, epithelial cells (or supernatant culture) were analysed by ELISA, co-immunoprecipitation or Western blot.

### Enzyme-linked immunosorbent assay (ELISA)

IL-8 secretion of A549 cell cultures was analysed by sandwich ELISA. After 24 h of incubation with PAM_3_CSK_4_ or *P. brasiliensis* yeasts, A549 cell culture supernatants were collected, centrifuged to remove fungi or cell debris, and then analysed using Duo Set Kit (R&D Systems/Bio-Techne, MN, USA), according to manufacturer’s instructions.

### Western blot

After *P. brasiliensis*-A549 cell interaction for 5 or 24 h, cells were washed with PBS, collected using a cell scraper, and incubated with RIPA lysis buffer (25 mM Tris–HCl pH 7.4 containing 150 mM NaCl, 0.1% sodium dodecyl sulphate—SDS-, 1% Triton X-100 and 0.5% sodium deoxycholate) containing protease inhibitors as previously described by Barros and co-workers (2016) ^9^. After 30 min at 4 °C, samples were centrifuged, and the resultant supernatant containing proteins was quantified using QuantiPro BCA Assay kit (Sigma-Aldrich/Merck, MO, USA), according to manufacturer’s instructions. Twenty μg of protein extract was solubilised in sample buffer (250 mM Tris–HCl pH 6.8, 40% glycerol (w/v), 8% SDS, 0.1% bromophenol blue, and 10% β-mercaptoethanol), and then subjected to SDS–PAGE (SDS–polyacrylamide gel electrophoresis) ^31^. After electrophoresis, proteins were transferred to PVDF (polyvinylidene difluoride) membranes ^32^, which in turn were blocked with 5% non-fat milk (Cell Signaling Technology, MA, USA) in TBST (200 mM Tris–HCl pH 8.0 containing 0.1% Tween-20) (for α3 integrin or β-actin analysis) or with Milk Diluent Blocking solution (SeraCare, MA, USA) (for TLR2). Next, membranes were incubated at 4 °C with TBST containing 5% bovine serum albumin (BSA) (Sigma-Aldrich/Merck, MO, USA) and one of the following antibodies: (1) anti-TLR2 1:1000 (Cell Signaling Technology, MA, USA, #2229), (2) anti-α3 integrin 1:1000 (Santa Cruz Biotechnology, TX, USA, sc-374242), or (3) anti-β-actin 1:15,000 (Sigma-Aldrich/Merck, MO, USA, A5441). After 16 h, membranes were incubated with horseradish peroxidase-conjugated anti-mouse or anti-rabbit (1:2000, #7076 and #7074, respectively, Cell Signaling Technology, MA, USA) in 1% BSA in TBST for 1 h at room temperature. After each step, membranes were washed three times with TBST. Finally, membranes were incubated with SuperSignal West Pico Chemiluminescent Substrate (Thermo Fisher Scientific, MA, USA) and proteins were detected by using an imaging system (UVITEC Cambridge, UK). Densitometric analyses were performed for protein level quantification using Image J (FIJI).

As mentioned in figure legends and/or supplementary information, in Western blot assays, β-actin was used as loading protein control for blots. For this, before incubation with primary antibodies, PVDF membranes were cut between 75 and 50 kDa markers. The upper membranes were incubated with anti-α3 integrin or -TLR2 antibodies, and the bottom, with anti-β-actin antibodies. All experiments were performed at least three times to guarantee data reproducibility and a representative Western blot was shown in this article. For clarity, Western blot images were edited and presented in the main article. Bands of the same blot with the same exposure time were cut and joined into an edited image that was shown in the main paper. The original Western blot images were presented in the supplementary information file. Different exposure times of the PVDF membranes, obtained in the UVITEC imaging system, were presented as supplementary figures. In this manner, it may be verified that the results of edited figures correspond to the original images and the differences of α3 integrin or TLR2 levels were not dependent on the exposure time of the PVDF membrane. Therefore, all authors declare the veracity/reproducibility of the data provided in this article. Besides, Western blot images were edited only for succinctness in the main paper and did not compromise the results of original blots.

### Coimmunoprecipitation

Aliquots containing 500 µg of proteins, obtained as described in “Western blot” section, were incubated with 3 µg of anti-α3 integrin (Santa Cruz Biotechnology, TX, USA, sc-374242). After 16 h at 4 °C, protein A/G conjugated agarose beads were added and incubated at 4 °C. After 3 h, beads were washed with RIPA lysis buffer, resuspended in sample buffer (as described in “Western blot” section), boiled for 5 min and centrifuged. Supernatants were loaded onto SDS–PAGE gel and analysed by Western blot using anti-TLR2 antibodies.

### Small interfering RNA (siRNA)

A549 cells were grown in 6 or 24 well plates for 1 day and serum-starved for 5 h. Then, cells were transfected using a mix of Lipofectamine RNAiMAX Reagent and Silencer Select Pre-designed siRNA for TLR2 (s-169, Invitrogen/Thermo Fisher Scientific, CA, EUA) at the final concentration of 10 nM. Silencer Select Negative Control siRNA (4390843, Invitrogen/Thermo Fisher Scientific, CA, EUA), which has a sequence that does not target any gene, was used as a negative control. After 24 h of transfection, cells were washed three times with DMEM and incubated for 24 h with: (1) 5 µg/mL of TLR2/1 ligand, PAM_3_CSK_4_ or (2) *P. brasiliensis* yeasts (MOI = 2.5) and then, culture supernatants were collected. IL-8 concentrations were determined by sandwich ELISA. Concomitantly, A549 cells were collected and TLR2 and α3 integrin levels were analysed by Western blot.

### Cell viability

A549 cell viability was determined by MTT (3-[4,5-dimethylthiazol-2-yl]-2,5diphenyltetrazolium bromide) assay, as previously described by Maza and co-workers (2012) ^7^. For all experiments, cell viability was greater than 95%.

### Statistical analyses

Statistical analyses were performed using ANOVA or Student’s *t* test through GraphPad Prism Software (CA, USA). Values were considered statistically significant at *p* ≤ 0.01.

## Supplementary information

Supplementary Information

## References

[1] Shikanai-Yasuda MA (2017). Brazilian guidelines for the clinical management of paracoccidioidomycosis. Rev. Soc. Bras. Med. Trop..

[2] Griffiths J, Colombo AL, Denning DW (2019). The case for paracoccidioidomycosis to be accepted as a neglected tropical (fungal) disease. PLoS Negl. Trop. Dis..

[3] Millington, M. A. *et al.* Paracoccidioidomicose: abordagem histórica e perspectivas de implantação da vigilância e controle. *Epidemiol. e Serv. saude Rev. do Sist. Unico Saude do Bras.* **27,** e0500002 (2018). 10.5123/S1679-49742018000500002 30133689

[4] Vieira, G. de D., Alves, T. da C., de Lima, S. M. D., Camargo, L. M. A. & de Sousa, C. M. Paracoccidioidomycosis in a western Brazilian Amazon State: Clinical-epidemiologic profile and spatial distribution of the disease. *Rev. Soc. Bras. Med. Trop.* **47,** 63–68 (2014). 10.1590/0037-8682-0225-2013 24603739

[5] Do Valle, A. C. F. *et al.* Paracoccidioidomycosis after highway construction, Rio de Janeiro, Brazil. *Emerg. Infect. Dis.* **23,** 1917–1919 (2017). 10.3201/eid2311.170934 PMC5652422 29048286

[6] Leiva-Juárez MM, Kolls JK, Evans SE (2018). Lung epithelial cells: therapeutically inducible effectors of antimicrobial defense. Mucosal Immunol..

[7] Maza, P. K. *et al. Paracoccidioides brasiliensis* induces secretion of IL-6 and IL-8 by lung epithelial cells. Modulation of host cytokine levels by fungal proteases. *Microbes Infect.* **14,** 1077–1085 (2012). 10.1016/j.micinf.2012.05.016 22687715

[8] Alcantara, C., Maza, P. K., Barros, B. C. S. C. & Suzuki, E. Role of protein kinase C in cytokine secretion by lung epithelial cells during infection with *Paracoccidioides brasiliensis*. *Pathog. Dis.* **73,** ftv045 (2015). 10.1093/femspd/ftv045 PMC4626587 26152710

[9] Barros BCSC, Maza PK, Alcantara C, Suzuki E (2016). *Paracoccidioides brasiliensis* induces recruitment of α3 and α5 integrins into epithelial cell membrane rafts, leading to cytokine secretion. Microbes Infect..

[10] Maza PK, Suzuki E (2016). *Histoplasma capsulatum*-induced cytokine secretion in lung epithelial cells is dependent on host integrins, Src-family kinase activation, and membrane raft recruitment. Front. Microbiol..

[11] de Oliveira P (2017). *Paracoccidioides brasiliensis* induces cytokine secretion in epithelial cells in a protease-activated receptor-dependent (PAR) manner. Med. Microbiol. Immunol..

[12] Almeida BR, Barros BCSC, Araújo ACL, Alcantara C, Suzuki E (2019). *Paracoccidioides* species present distinct fungal adherence to epithelial lung cells and promote different IL-8 secretion levels. Med. Microbiol. Immunol..

[13] Alcantara C (2020). *Histoplasma capsulatum* chemotypes I and II induce IL-8 secretion in lung epithelial cells in distinct manners. Med. Mycol..

[14] Williams PB (2016). Innate and adaptive immune response to fungal products and allergens. J. Allergy Clin. Immunol. Pract..

[15] Taghavi M, Khosravi A, Mortaz E, Nikaein D, Athari SS (2017). Role of pathogen-associated molecular patterns (PAMPs) in immune responses to fungal infections. Eur. J. Pharmacol..

[16] Marre ML, Petnicki-Ocwieja T, Defrancesco AS, Darcy CT, Hu LT (2010). Human integrin α3β1 regulates TLR2 recognition of lipopeptides from endosomal compartments. PLoS ONE.

[17] Lerman YV (2014). Sepsis lethality via exacerbated tissue infiltration and TLR-induced cytokine production by neutrophils is integrin α3β1-dependent. Blood.

[18] Regueiro V (2009). *Klebsiella pneumoniae* increases the levels of toll-like receptors 2 and 4 in human airway epithelial cells. Infect. Immun..

[19] Jin MS (2007). Crystal structure of the TLR1-TLR2 heterodimer induced by binding of a tri-acylated lipopeptide. Cell.

[20] Patin EC, Thompson A, Orr SJ (2019). Pattern recognition receptors in fungal immunity. Semin. Cell Dev. Biol..

[21] Bourgeois C, Kuchler K, Gekara N, Aucoin DP (2012). Fungal pathogens- a sweet and sour treat for toll-like receptors. Front. Cell. Infect. Microbiol..

[22] Alegre-Maller ACP (2014). Therapeutic administration of recombinant paracoccin confers protection against *Paracoccidioides brasiliensis* infection: Involvement of TLRs. PLoS Negl. Trop. Dis..

[23] Balderramas, H. A. *et al.* Human neutrophils produce IL-12, IL-10, PGE2 and LTB4 in response to *Paracoccidioides brasiliensis*. Involvement of TLR2, mannose receptor and dectin-1. *Cytokine* **67,** 36–43 (2014). 10.1016/j.cyto.2014.02.004 24680480

[24] Loures FV, Pina A, Felonato M, Calich VLG (2009). TLR2 is a negative regulator of Th17 cells and tissue pathology in a pulmonary model of fungal infection. J. Immunol..

[25] Gianni T, Campadelli-Fiume G (2014). The epithelial αvβ3-integrin boosts the MYD88-dependent TLR2 signaling in response to viral and bacterial components. PLoS Pathog..

[26] Hsu CC (2016). Snake venom disintegrin inhibits the activation of Toll-Like Receptors and alleviates sepsis through integrin alphaVbeta3 blockade. Sci. Rep..

[27] McGarry T (2015). Toll-like receptor 2 (TLR2) induces migration and invasive mechanisms in rheumatoid arthritis. Arthritis Res. Ther..

[28] Kim KK (2009). Epithelial cell α3β1 integrin links β-catenin and Smad signaling to promote myofibroblast formation and pulmonary fibrosis. J. Clin. Invest..

[29] Benard G (2005). Contribution to the natural history of paracoccidioidomycosis: Identification of the primary pulmonary infection in the severe acute form of the disease: a case report. Clin. Infect. Dis..

[30] Ywazaki CY, Maza PK, Suzuki E, Takahashi HK, Straus AH (2011). Role of host glycosphingolipids on *Paracoccidioides brasiliensis* adhesion. Mycopathologia.

[31] Laemmli UK (1970). Cleavage of structural proteins during the assembly of the head of bacteriophage T4. Nature.

[32] Maza PK, Straus AH, Toledo MS, Takahashi HK, Suzuki E (2008). Interaction of epithelial cell membrane rafts with *Paracoccidioides brasiliensis* leads to fungal adhesion and Src-family kinase activation. Microbes Infect..

