# Supplementary material for: Paracoccidioides brasiliensis downmodulates α3 integrin levels in human lung epithelial cells in a TLR2-dependent manner
Source: Sci Rep. 2020 Nov 10;10:19483. doi: 10.1038/s41598-020-76557-6 (PMC7655819; doi:10.1038/s41598-020-76557-6)
Supplement: Supplementary file 1 — Supplementary Information [file 41598_2020_76557_MOESM1_ESM.pdf]

Paracoccidioides brasiliensis downmodulates α3 integrin levels in human lung epithelial cells in a TLR2-dependent manner

Bianca Carla Silva Campitelli de Barros[1], Bruna Rocha Almeida[1], Erika Suzuki[1]*.

Escola Paulista de Medicina, Universidade Federal de São Paulo, Department of Microbiology, Immunology and Parasitology, São Paulo, 04023-062, Brazil

***Corresponding author: erika.suzuki@unifesp.br**

Supplementary Information

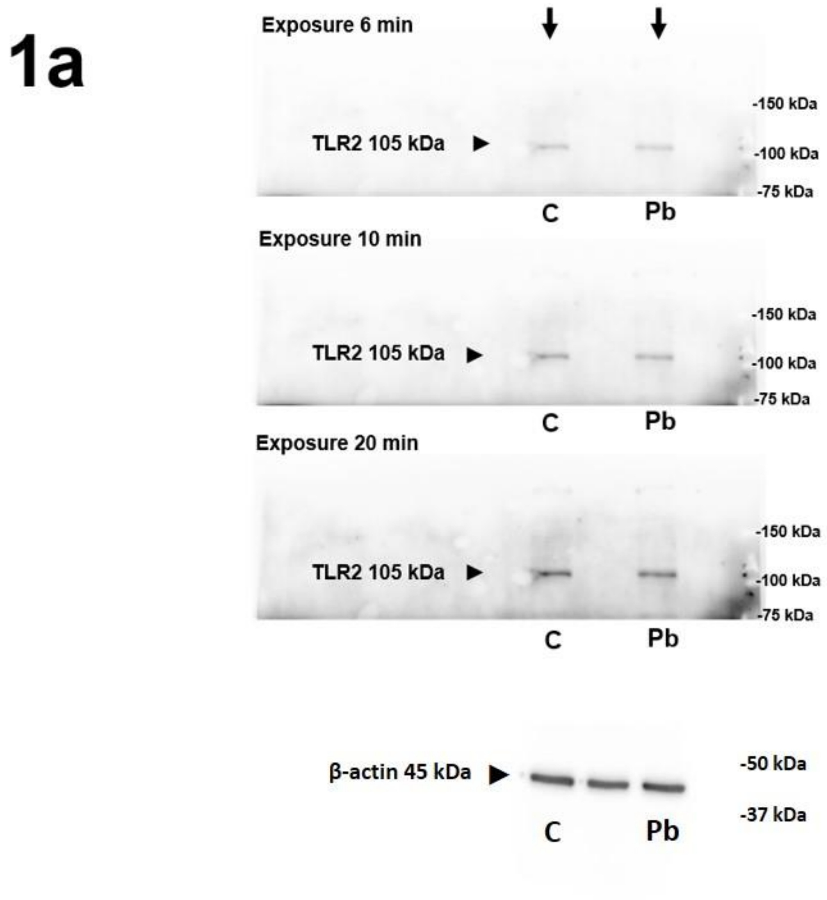

Supplementary Figure 1. Images of multiple exposure times of Western blot membranes showed in Figure 1a

For membrane 1a, it was used a 6% polyacrylamide gel. To evaluate β-actin, membrane 1a was cut in order to analyse only samples with the observed TLR2 bands.

For this membrane, it was used the molecular weight marker "Precision plus protein standards dual color" (BIO-RAD code #161-0374).

To analyse β-actin, PVDF membrane was cut between the 75 kDa and 50 kDa markers.

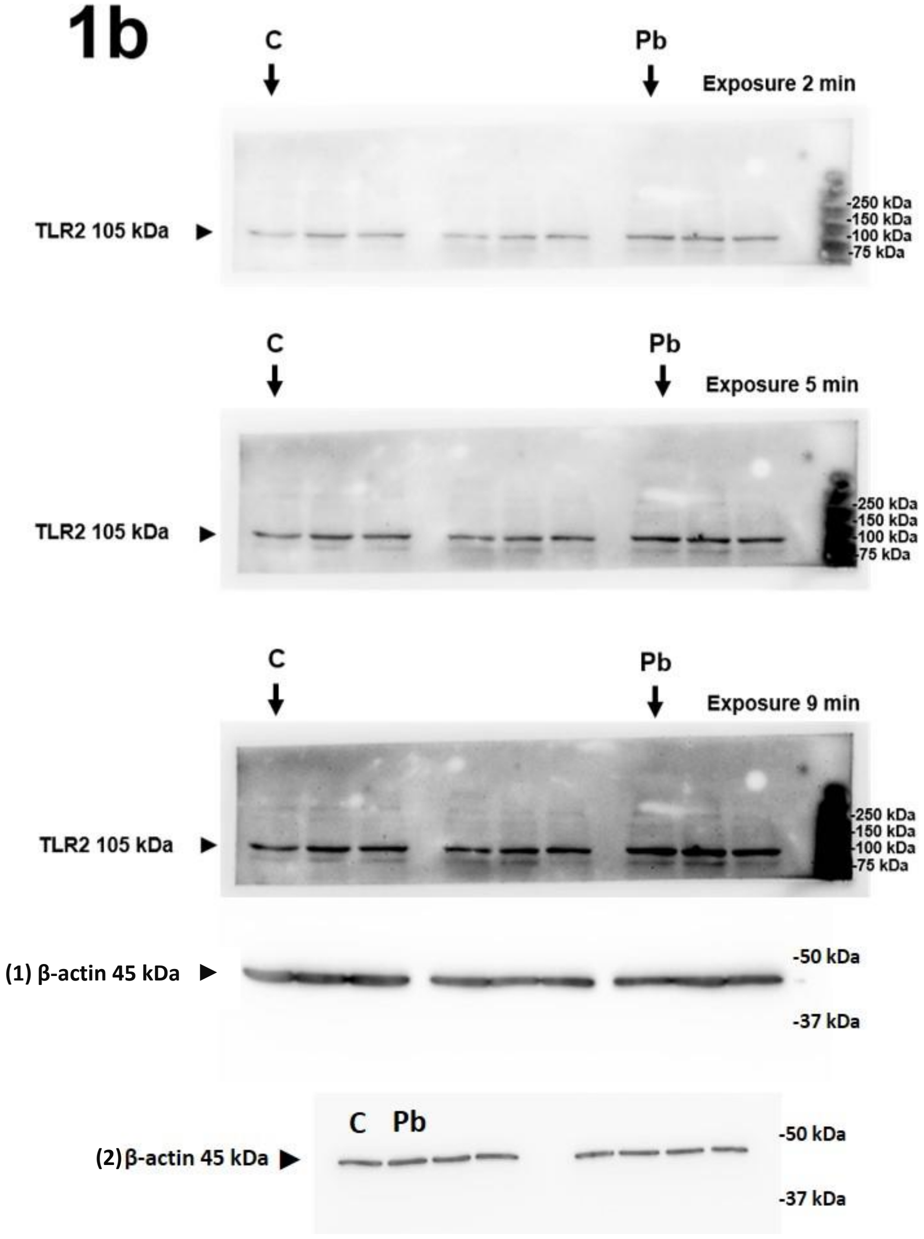

Continuation of the Supplementary Figure 1. Images of multiple exposure times of Western blot membranes showed in Figure 1b

For membrane 1b, it was used a 10% polyacrylamide gel.

As we did not observe separate β-actin bands in the original membrane [(1) β-actin 45 kDa], we submitted aliquots of some of these samples to another β-actin analysis by Western blot [(2) β-actin 45 kDa].

For these membranes, it was used the molecular weight marker “Precision plus protein standards dual color” (BIO-RAD code #161-0374).

To analyse β-actin**,** PVDF membranes were cut between the 75 kDa and 50 kDa markers.

1c

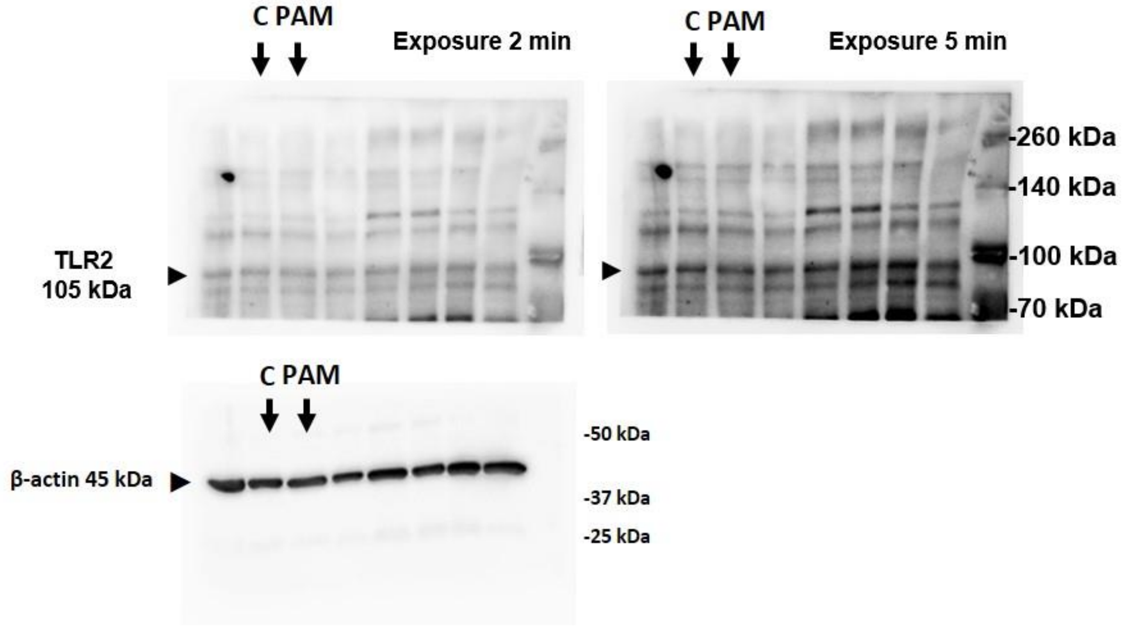

1d

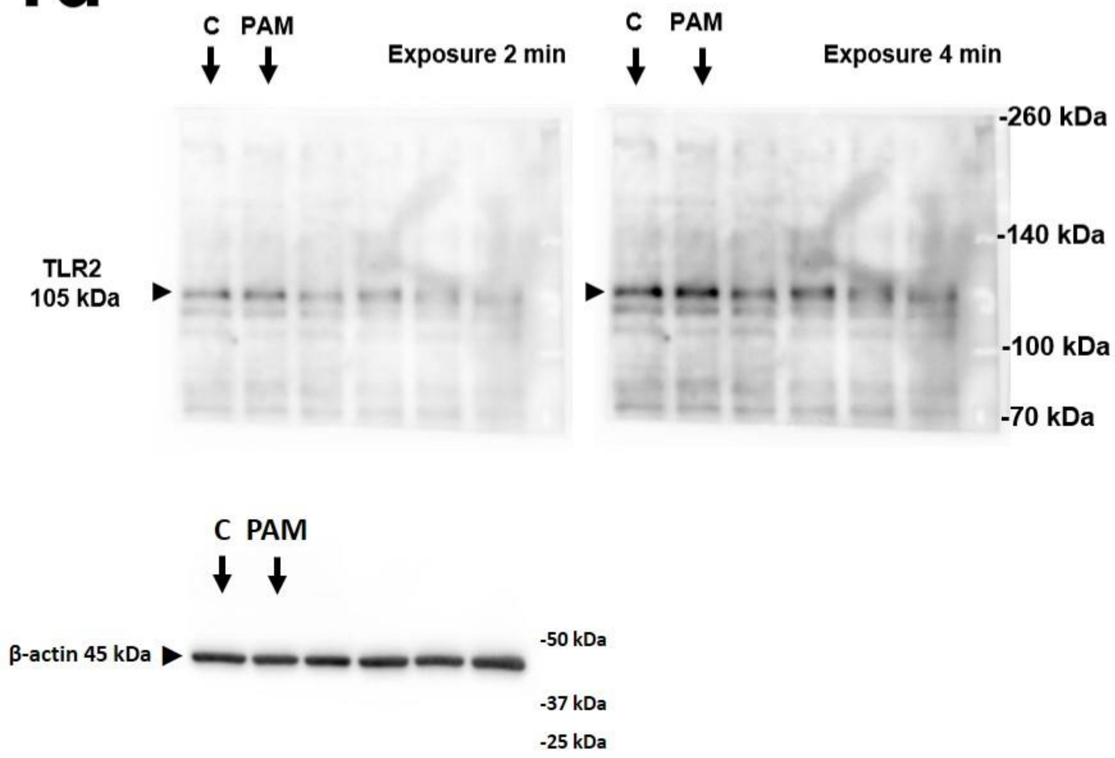

Continuation of the Supplementary Figure 1. Images of multiple exposure times of Western blot membranes showed in Figures 1c and 1d

For membrane 1c, it was used an 8% polyacrylamide gel.

For membrane 1d, it was used a 6% polyacrylamide gel.

For both membranes, it was used the molecular weight marker “Spectra Multicolor broad range protein ladder” (Thermo Scientific code #26634).

To analyse β-actin, PVDF membranes were cut near the 70 kDa and 50 kDa markers.

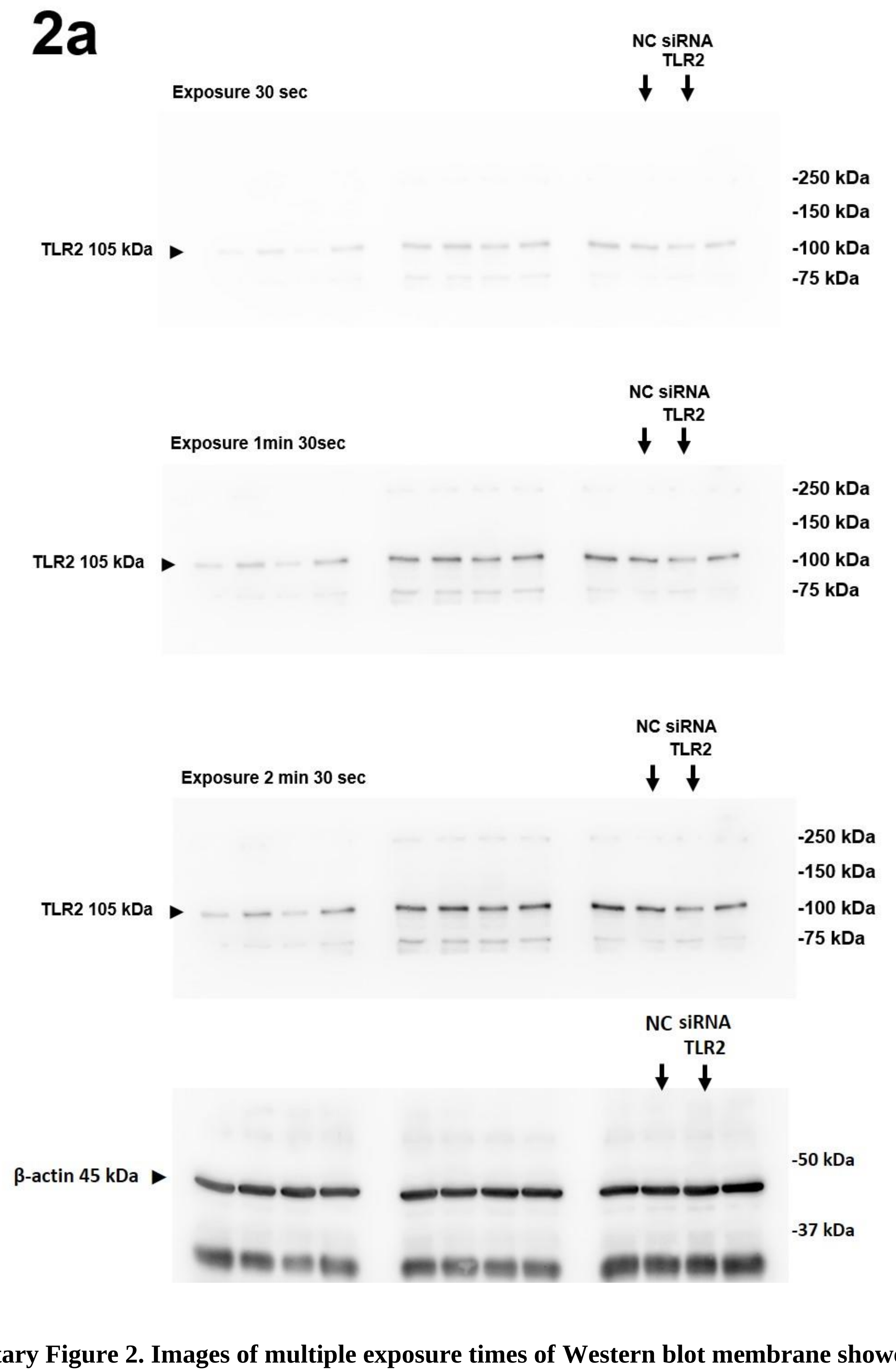

Supplementary Figure 2. Images of multiple exposure times of Western blot membrane showed in Figure 2a

For this membrane, it was used an 8% polyacrylamide gel.

It was used the molecular weight marker “Precision plus protein standards dual color” (BIO-RAD code #161-0374).

To analyse β-actin, PVDF membrane was cut between the 75 kDa and 50 kDa markers.

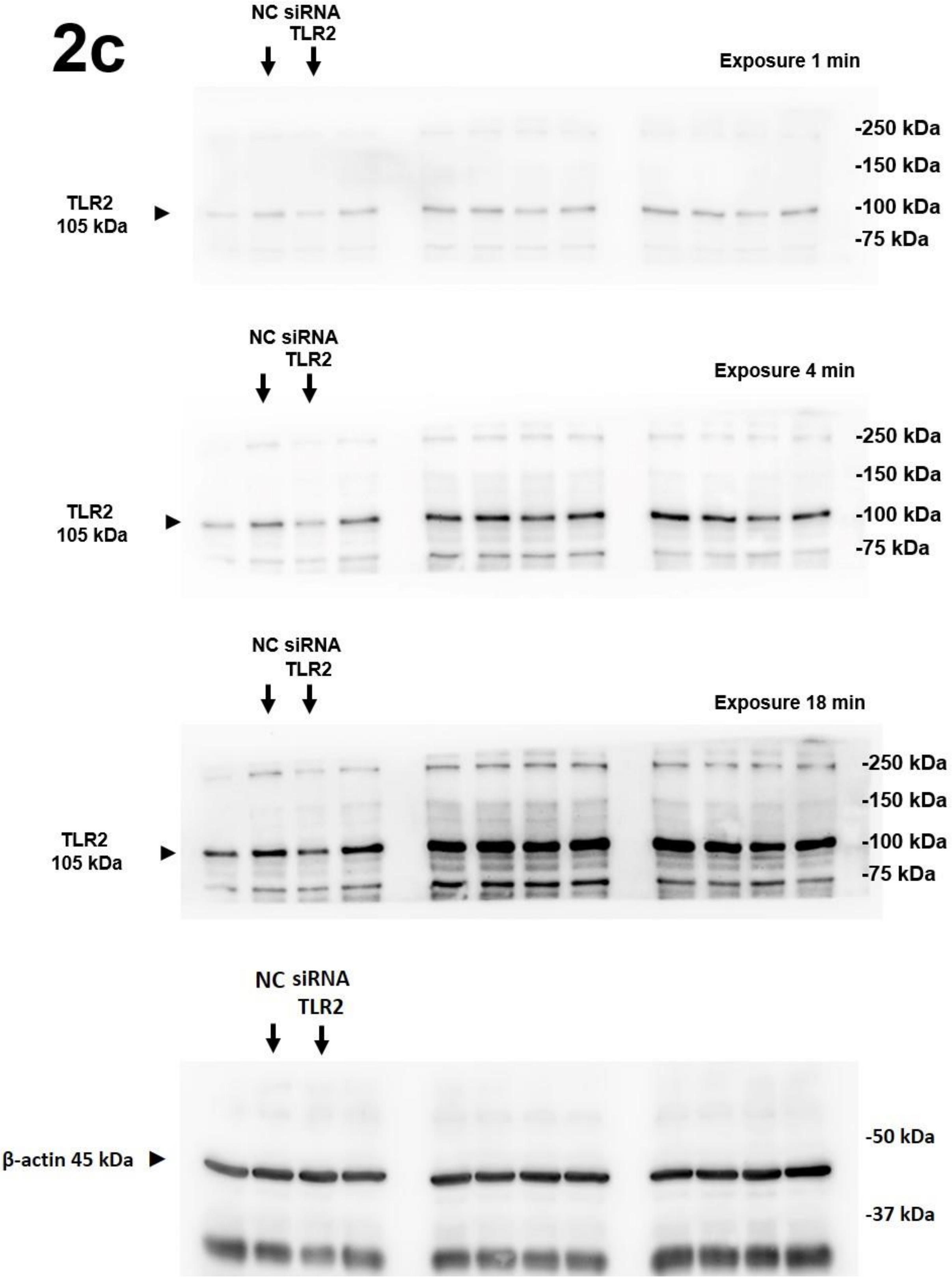

Continuation of the Supplementary Figure 2. Images of multiple exposure times of Western blot membrane showed in Figure 2c

For this membrane, it was used an 8% polyacrylamide gel.

It was used the molecular weight marker “Precision plus protein standards dual color” (BIO-RAD code #161-0374).

To analyse β-actin, PVDF membrane was cut between the 75 kDa and 50 kDa markers.

3a

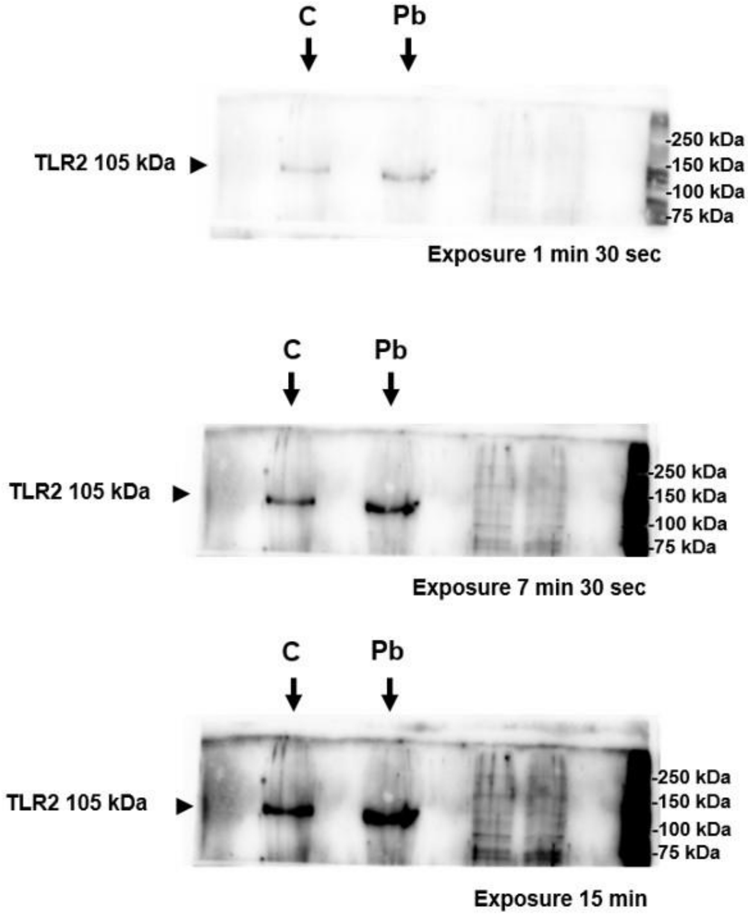

3b

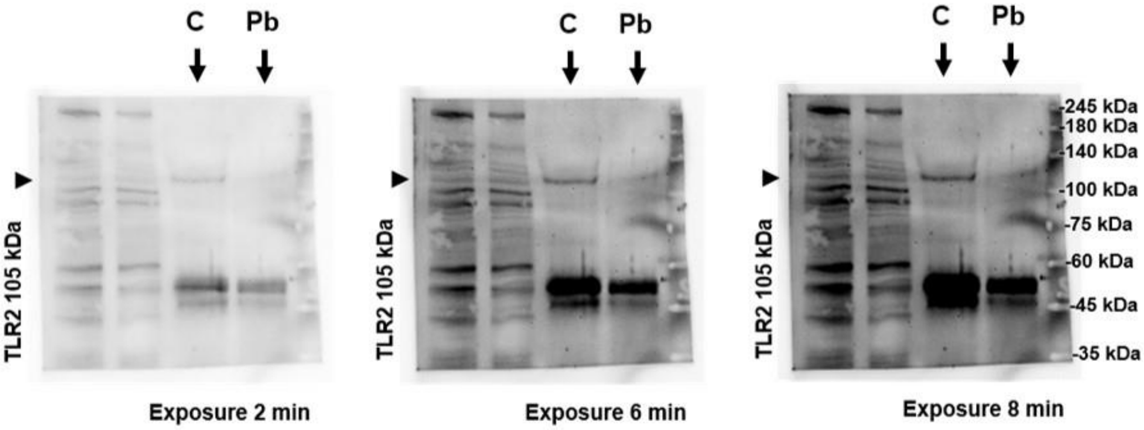

Supplementary Figure 3. Images of multiple exposure times of Western blot membranes showed in Figures 3a and 3b

For membrane 3a, it was used a 10% polyacrylamide gel and the molecular weight marker “Precision plus protein standards dual color” (BIO-RAD code #161-0374).

For membrane 3b, it was used a 6% polyacrylamide gel and the molecular weight marker “TrueColor High Range Protein Marker” (Sinapse Inc code #S2600).

Membrane 3a was cut near the 75 kDa marker before incubation with TLR2 antibody.

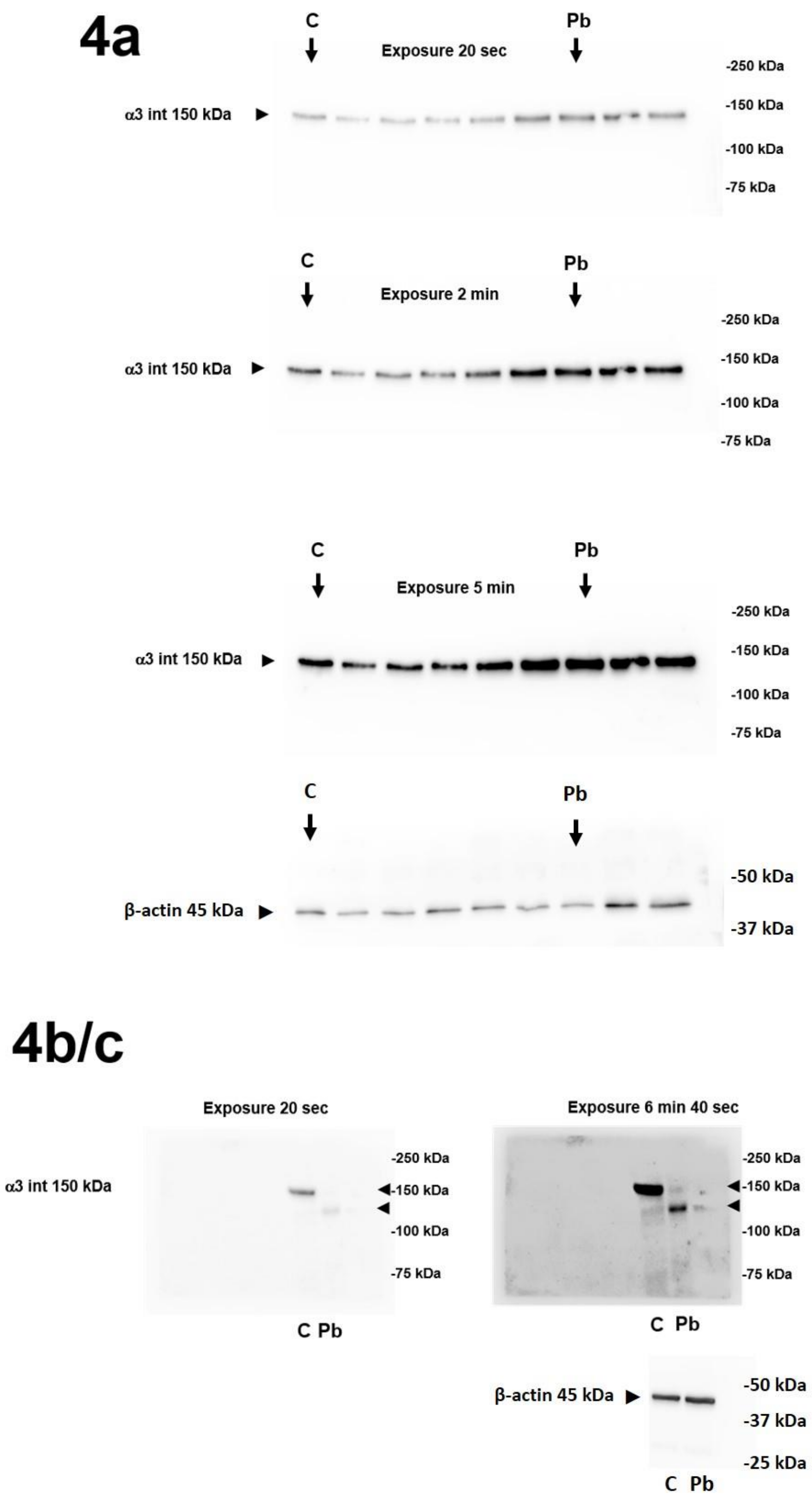

Supplementary Figure 4. Images of multiple exposure times of Western blot membranes showed in Figures 4a, 4b and 4c

For these membranes, it was used a 6% polyacrylamide gel.

It was used the molecular weight marker "Precision plus protein standards dual color" (BIO-RAD code #161-0374).

To analyse β-actin**,** PVDF membranes were cut between the 75 kDa and 50 kDa markers.

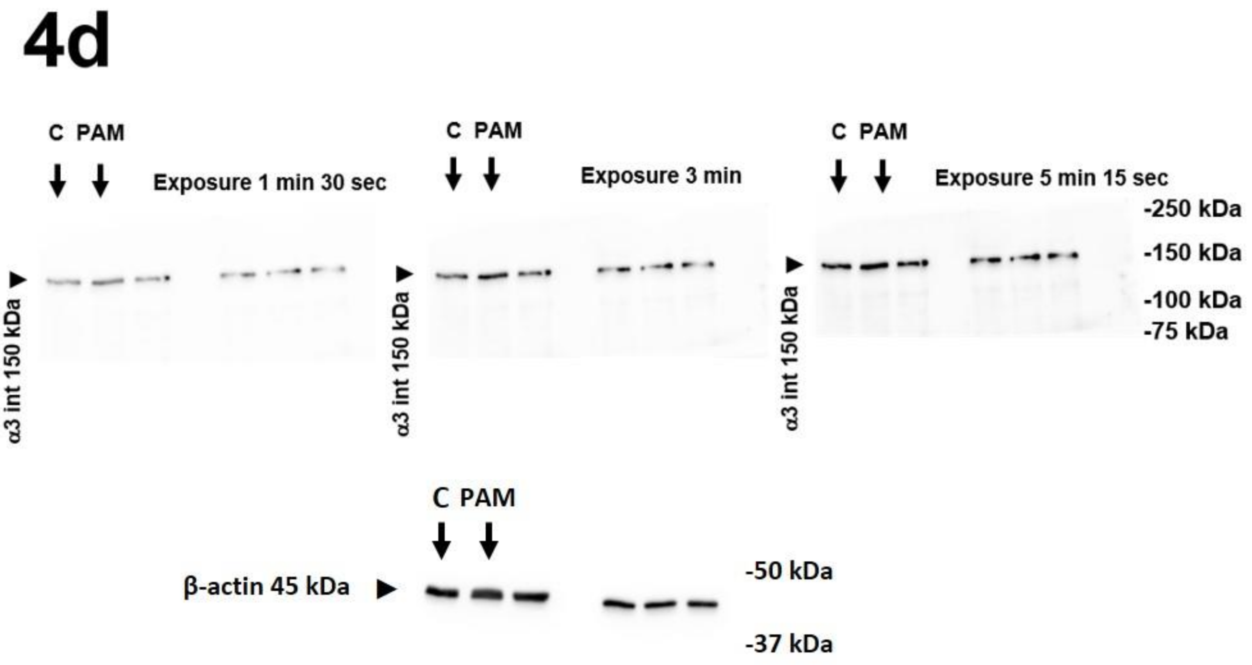

Continuation of the Supplementary Figure 4. Images of multiple exposure times of Western blot membranes showed in Figure 4d

For membrane 4d, it was used a 6% polyacrylamide gel.

It was used the molecular weight marker “Precision plus protein standards dual color” (BIO-RAD code #161-0374).

To analyse β-actin, PVDF membrane was cut between 75 kDa and 50 kDa.

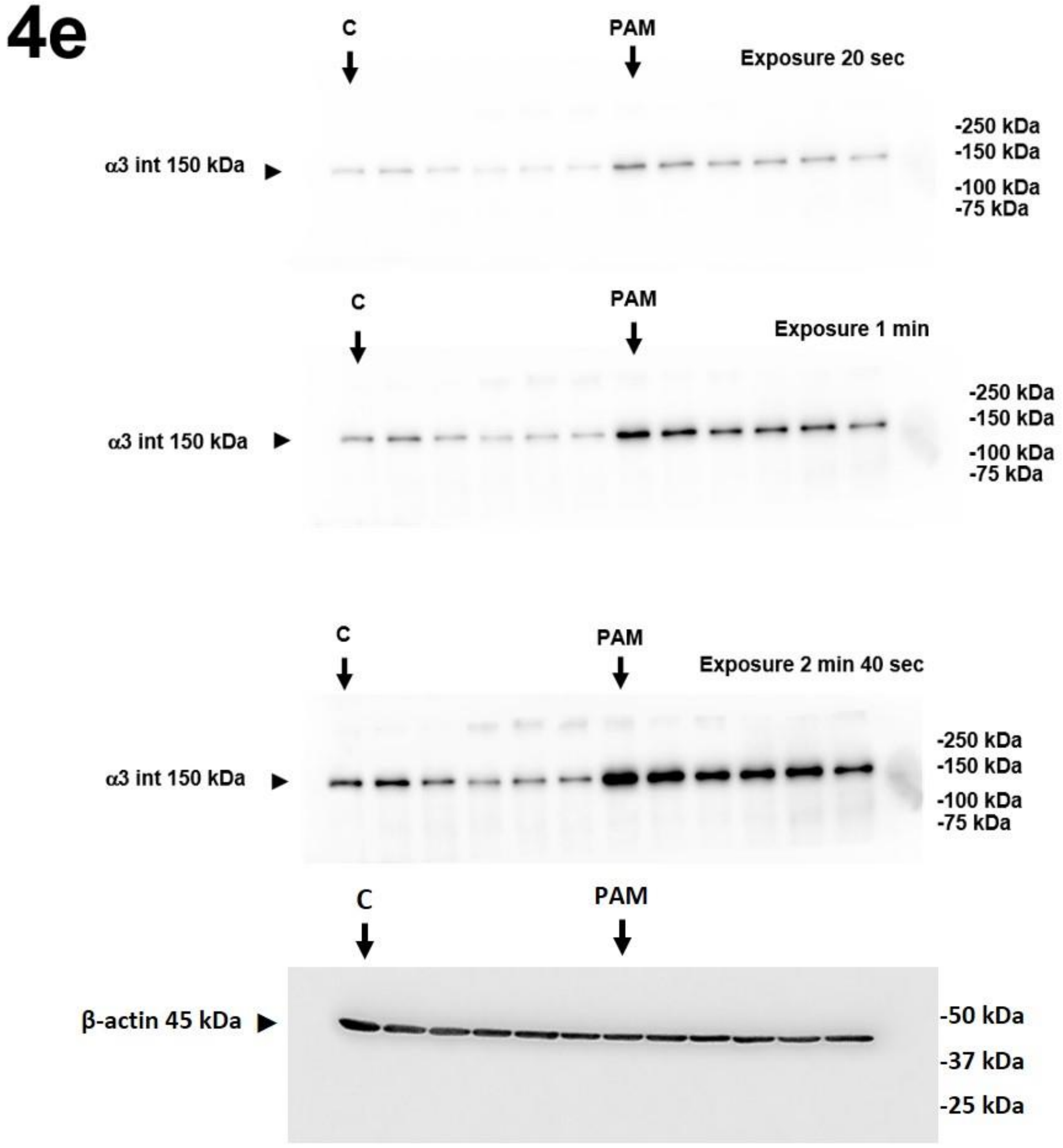

Continuation of the Supplementary Figure 4. Images of multiple exposure times of Western blot membranes showed in Figure 4e

For membrane 4e, it was used a 10% polyacrylamide gel.

It was used the molecular weight marker "Precision plus protein standards dual color" (BIO-RAD code #161-0374).

To analyse β-actin, PVDF membrane was cut between 75 kDa and 50 kDa.

5a

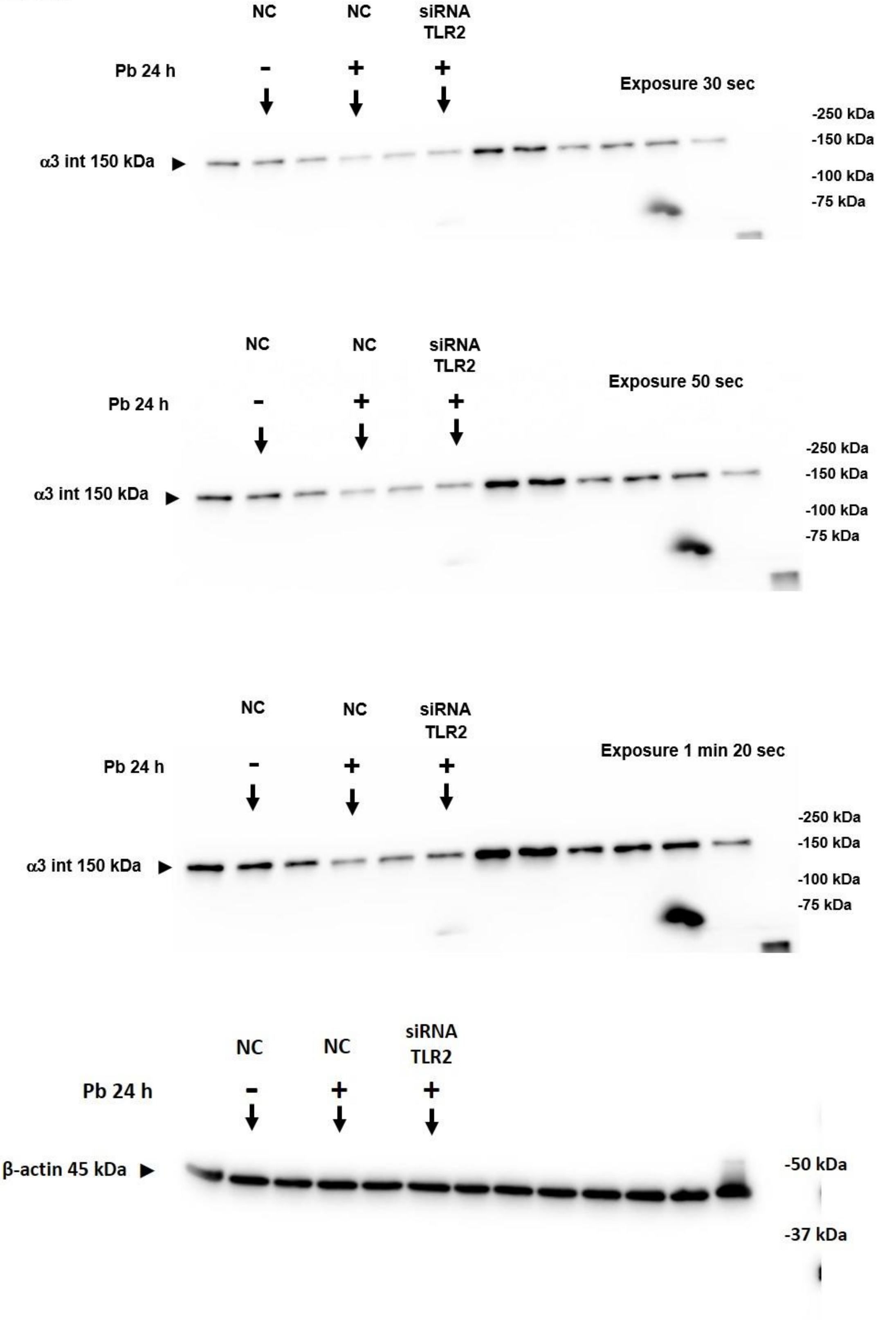

Supplementary Figure 5. Images of multiple exposure times of Western blot membrane showed in Figure 5a

For this membrane, it was used a 10% polyacrylamide gel.

It was used the molecular weight marker "Precision plus protein standards dual color" (BIO-RAD code #161-0374).

To analyse β-actin, PVDF membranes were cut between 75 kDa and 50 kDa.

5b

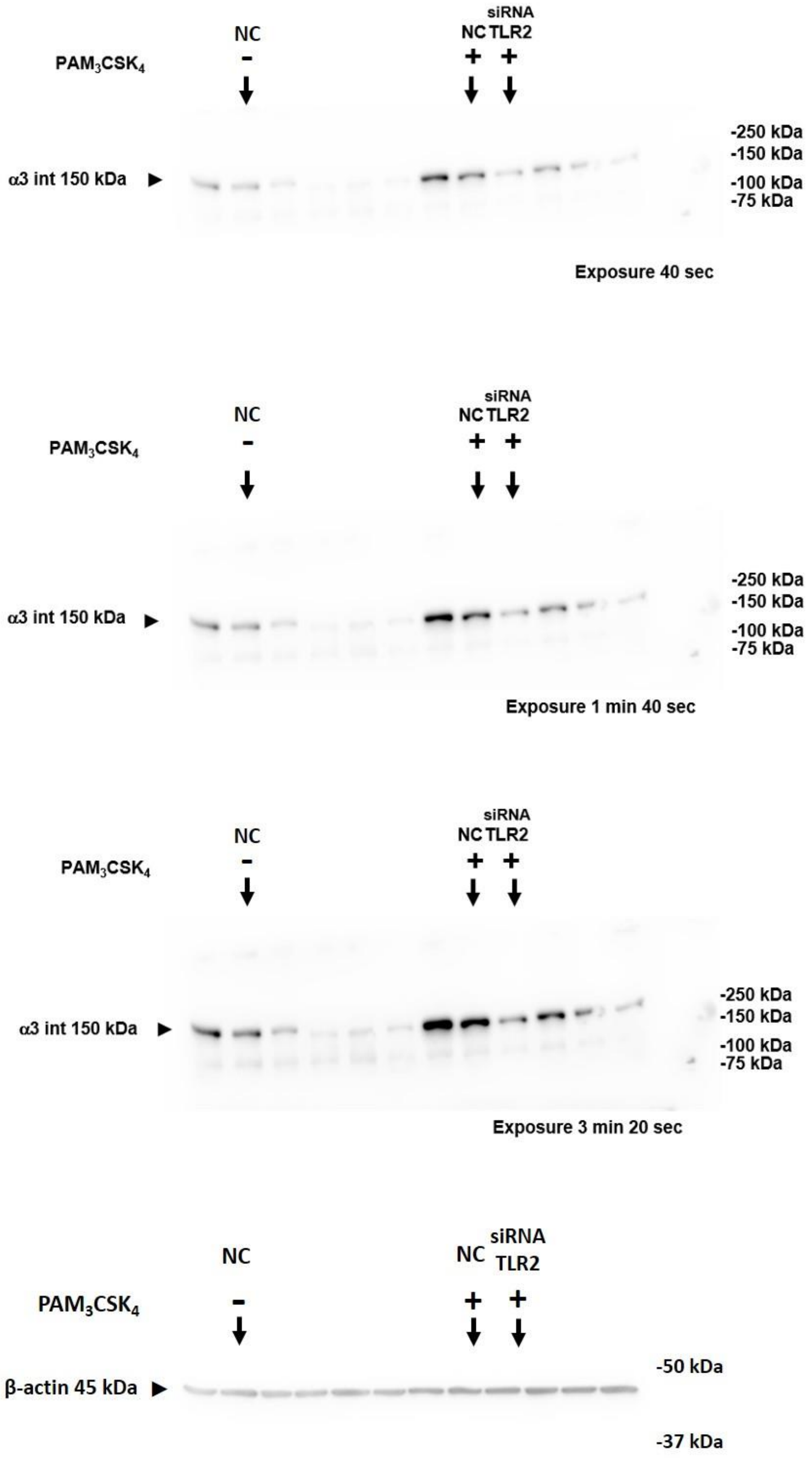

Continuation of the Supplementary Figure 5. Images of multiple exposure times of Western blot membrane showed in Figure 5b

For this membrane, it was used a 10% polyacrylamide gel.

It was used the molecular weight marker “Precision plus protein standards dual color” (BIO-RAD code #161-0374).

To analyse β-actin, PVDF membranes were cut between 75 kDa and 50 kDa.

6

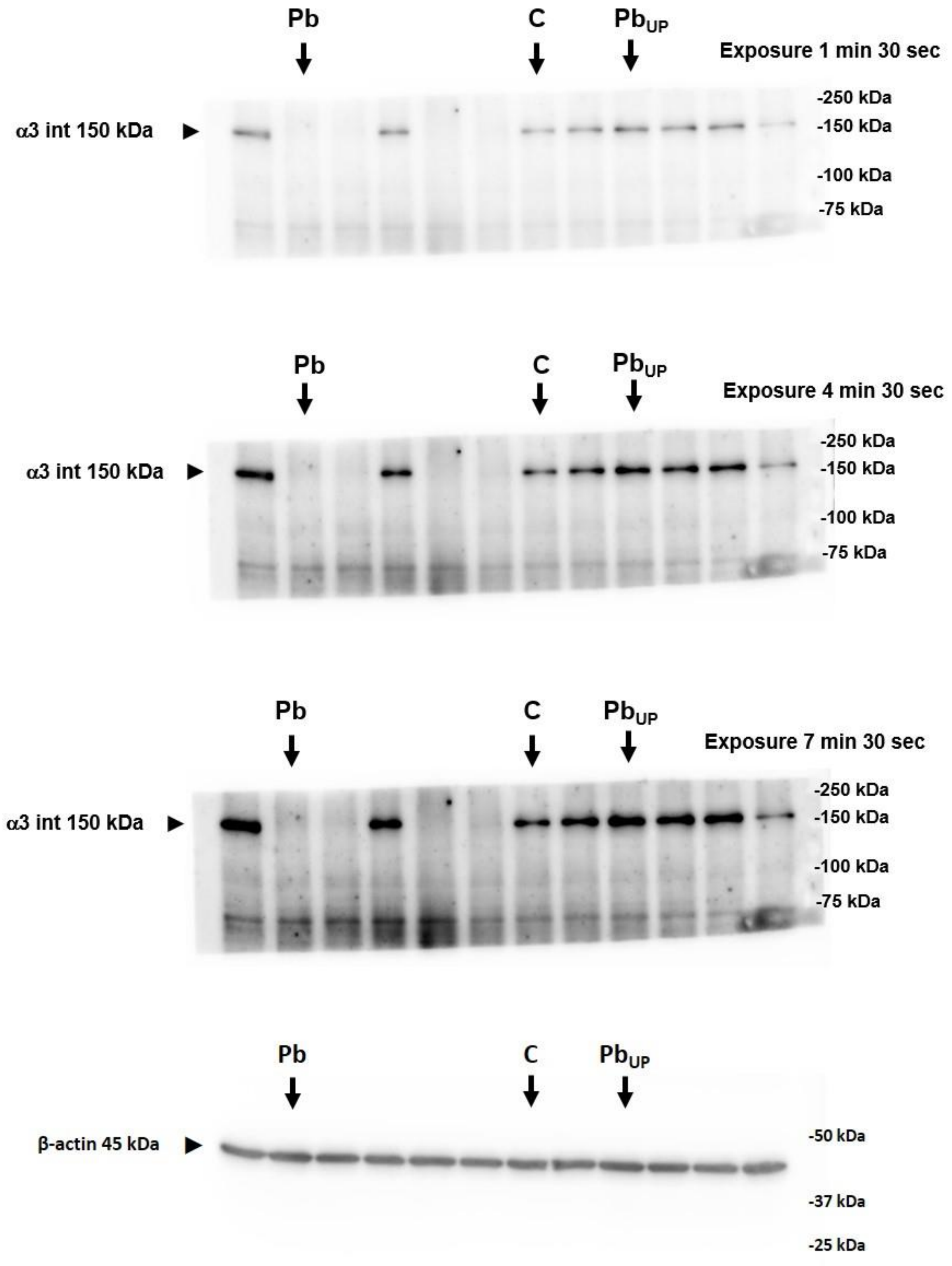

Supplementary Figure 6. Images of multiple exposure times of Western blot membrane showed in Figure 6

For this membrane, it was used a 10% polyacrylamide gel.

It was used the molecular weight marker “Precision plus protein standards dual color” (BIO-RAD code #161-0374).

To analyse β-actin, PVDF membranes were cut between 75 kDa and 50 kDa.
